# Improved epileptic seizure detection combining dynamic feature normalization with EEG novelty detection

**DOI:** 10.1007/s11517-016-1479-8

**Published:** 2016-04-06

**Authors:** J. G. Bogaarts, D. M. W. Hilkman, E. D. Gommer, V. H. J. M. van Kranen-Mastenbroek, J. P. H. Reulen

**Affiliations:** Department of Clinical Neurophysiology, MUMC+, P. Debyelaan 25, 6229 HX Maastricht, The Netherlands

**Keywords:** Seizure detection, SVM, PCA, EEG, Feature normalization

## Abstract

Continuous electroencephalographic monitoring of critically ill patients is an established procedure in intensive care units. Seizure detection algorithms, such as support vector machines (SVM), play a prominent role in this procedure. To correct for inter-human differences in EEG characteristics, as well as for intra-human EEG variability over time, dynamic EEG feature normalization is essential. Recently, the median decaying memory (MDM) approach was determined to be the best method of normalization. MDM uses a sliding baseline buffer of EEG epochs to calculate feature normalization constants. However, while this method does include non-seizure EEG epochs, it also includes EEG activity that can have a detrimental effect on the normalization and subsequent seizure detection performance. In this study, EEG data that is to be incorporated into the baseline buffer are automatically selected based on a novelty detection algorithm (Novelty-MDM). Performance of an SVM-based seizure detection framework is evaluated in 17 long-term ICU registrations using the area under the sensitivity–specificity ROC curve. This evaluation compares three different EEG normalization methods, namely a fixed baseline buffer (FB), the median decaying memory (MDM) approach, and our novelty median decaying memory (Novelty-MDM) method. It is demonstrated that MDM did not improve overall performance compared to FB (*p* < 0.27), partly because seizure like episodes were included in the baseline. More importantly, Novelty-MDM significantly outperforms both FB (*p* = 0.015) and MDM (*p* = 0.0065).

**Electronic supplementary material:**

The online version of this article (doi:10.1007/s11517-016-1479-8) contains supplementary material, which is available to authorized users.

## Introduction

Nowadays, continuous electroencephalographic monitoring (cEEG) of critically ill patients is an established procedure in intensive care units (ICU). Quantitative EEG (qEEG) allows many hours of EEG data to be compressed onto a graph, greatly reducing the amount of time necessary to detect seizures and transient EEG changes [18]. Up to 48 % of ICU patients experience non-convulsive seizures (NCS), much more than would be detected by clinical observation alone [5, 8, 23, 26, 32]. If those seizures are not detected, and treatment is thus not offered, those patients may suffer brain damage. Therefore, automated seizure detection methods based on qEEG would be an important addition to the cEEG procedure. In past years, numerous seizure detection methods have been developed [1, 2, 7, 12, 21, 24, 29]. Research has mainly focused on two aspects of automatic seizure detection: EEG feature computation and methodological aspects of classification [24]. Due to time-varying EEG dynamics and high variability in EEG characteristics between patients, reliable automated seizure detection is difficult [15]. Because of this, feature normalization is essential for patient-independent epileptic seizure detection. Some research has evaluated the influence of non-stationary EEG background activity on seizure detection [3, 20]. We recently introduced a feature baseline correction (FBC) procedure which reduces inter-patient variability by correcting for differences in background EEG characteristics [3]. FBC is a feature normalization method based on visual inspection wherein a seizure-free EEG segment is selected at the start of a monitoring session. However, during long-term cEEG, background EEG changes and differences between different patients can be of an equivalent magnitude. Therefore, feature normalization should be applied in a dynamic time-dependent manner [20]. EEG baseline variation may be due to circadian rhythm, changes in the state of the patient’s vigilance, a response to medication, or changes in EEG recording quality, e.g. changing electrode-tissue impedances [9, 15]. For optimal FBC functioning, the non-seizure EEG baseline segment needs to be updated to adapt to changes in the background EEG. Logesparan et al. [20] showed that a normalization procedure based on median decaying memory (MDM) might be a promising normalization method. It computes a feature normalization factor N_F_, based on an on-going unsupervised update of the baseline EEG buffer. However, during long-term cEEG, artefacts, short-duration epileptiform events, and multiple successive seizures can be numerous. Because if this unsupervised baseline updated might result in corrupt N_F_ calculation and by that hamper seizure detection performance, this raises the question of whether unsupervised MDM is robust enough when cEEG conditions are less than optimal. Our hypothesis is that a semi-supervised baseline update may significantly improve MDM performance. The improvement our MDM approach offers is based upon our method’s ability to automatically reject EEG epochs from the baseline buffer. To accomplish this, we implement a novelty detection algorithm. Novelty detection classifies test data that differ in some respect from data available during training [22]. A novelty detector trained on the current baseline segment is used to detect ‘Novel’ epochs. These epochs differ significantly from the current baseline epochs and thus most likely contain artefacts or EEG patterns not similar to actual baseline EEG activity. Consequently, only epochs classified as ‘Non-Novel’ are used to update the baseline buffer. With this updated baseline buffer, the feature normalization factor and novelty detector are updated. Our major aim is to evaluate the effect of MDM feature normalization, with and without ‘Novelty Detection’, on support vector machine (SVM)-based seizure detection performance. Seizure detection performance is evaluated in terms of the area under the receiver operator characteristics (ROC) curve. To complete our study, the standard fixed baseline method [3] is compared to MDM and Novelty-MDM.

## Materials and methods

### EEG test dataset

The test dataset consists of 53 cEEG registrations recorded as part of an ongoing ICU monitoring study. At our hospital’s general ICU, patients in a comatose state due to central neurological damage (GSC ≤ 8) were prospectively enrolled in a non-blinded, non-randomized observational study between January 2011 and March 2014. This study was approved by our hospital’s ethical committee. With informed consent, and permission from each patient’s legal representatives, cEEG was performed. To be included in this study, patients had to have been admitted to the ICU, be 18 years of age or older, have central neurological damage and be in a coma (GCS ≤ 8), and EEG electrode placement had to be feasible. Patients were selected after consulting the neurologist or neurosurgeon responsible for their treatment. During their EEG registration, 17 out of the 53 patients experienced convulsive and/or non-convulsive seizures due to various aetiologies (Table 1). The total number of seizures in the dataset was 1362 and varied per patient from 1 to 384 with a median number of 49 seizures. The minimal duration of a seizure to be annotated as such was 10 s, as is recommended by the International Federation of Clinical Neurophysiology [6]. The total duration of EEG registration was 4018 h (median duration: 66 h, range 5–210 h). EEG registration was stopped as soon as GCS dropped below 8. EEG electrode configuration was done according to the international 10–20 electrode configuration system, with 19 active electrodes. Signals were processed in the common average derivation. Seizure detection was performed retrospectively while simulating online detection.

**Table 1:** Patient characteristics

Patient #	Gender	Age	Monitoring duration (h)	Aetiology
ICM0006	Female	29	101.00	Not waking after surgery (aortic surgery)
ICM0007	Male	65	41.25	Aortic rupture
ICM0013	Female	57	52.00	Subarachnoid haemorrhage
ICM0015	Male	80	97.50	Trauma with subdural haematoma
ICM0016	Male	69	142.00	Trauma with subdural haematoma
ICM0019	Male	75	76.25	Postanoxic encephalopathy
ICM0021	Male	69	122.75	Status epilepticus
ICM0022	Female	70	29.00	Subarachnoid haemorrhage
ICM0028	Male	43	35.75	Status epilepticus
ICM0030	Male	81	67.00	Postanoxic encephalopathy
ICM0031	Male	66	190.00	Status epilepticus
ICM0034	Female	69	94.75	Postanoxic encephalopathy
ICM0042	Female	38	345.50	Status epilepticus
ICM0047	Male	20	236.75	Status epilepticus
ICM0048	Male	60	40.50	Trauma
ICM0051	Female	66	168.50	Postanoxic encephalopathy
ICM0053	Male	67	128.50	Postanoxic encephalopathy

### Feature extraction

EEG recordings were recorded with a sample frequency of 250 Hz, band-pass filtered between 0.5 and 32 Hz and subsequently down-sampled to 25 Hz. Each of the 19 EEG channels was then partitioned into 10-s epochs with a 5-s (50 %) overlap between epochs. From each epoch in each common-average referenced EEG channel, 103 quantitative features were extracted [13, 30]. These features stem from different signal description domains such as time, frequency, and information theory and are listed in Table 2. Each EEG epoch is now described by a 103 number long feature vector per channel.

**Table 2:** List of EEG features extracted for each single channel EEG epoch

EEG features
Total power (0–12 Hz)
Peak frequency of spectrum
Spectral edge frequency (SEF80 %, SEF90 %, SEF95 %)
Power in 2 Hz width subbands (0–2, 1–3,…10–12 Hz)
Normalized power in same subbands
Wavelet energy (Db4 wavelet coefficient corresponding to 1–2 Hz)
Curve length
Number of maxima and minima
Root mean square amplitude
Hjorth parameters (activity, mobility and complexity)
Zero crossing rate (ZCR), ZCR of the Δ and the ΔΔ
Variance of Δ and ΔΔ
Autoregressive modelling error (AR model order 1–9)
Skewness and Kurtosis
Nonlinear energy
Shannon entropy, spectral entropy, Singular value decomposition entropy
Fisher information
Linear filterbank: 15 subbands (0–2, 1–3, …14–16 Hz)
15 cepstral coefficients
15 second order frequency filtered bank energies
Peak–peak voltage

### Baseline buffer selection

At the start of each monitoring session, an EEG expert selected 3 min of artefact- and seizure-free EEG. This EEG segment served as a baseline buffer and was used to calculate feature baseline values (*F*_bsl_). Due to various aetiologies, this baseline EEG was allowed to contain non-seizure abnormalities such as periodic discharges, sharp spikes, or a burst-suppression pattern. To restore EEG signal quality, electrode maintenance was often necessary. Because EEG signals registered before electrode restoration can differ markedly from those registered after electrode restoration, a new 3-min baseline was manually chosen each time electrode maintenance was performed.

### MDM-based feature baseline update

The median decaying memory approach [20] is used to update a feature baseline value *F*_bsl_ for each feature in each channel separately. Updating consists of a weighted average of the median feature value F of the epochs currently in the buffer and the previous value of *F*_bsl_:

1 $$F_{\text{bsl}} (i) = (1 - \lambda ){\text{median}}(F(i - 1) \ldots F(i - K)) + \lambda F_{\text{bsl}} (i - 1)$$

Equation 1 has two free parameters: buffer size K and λ. Additional memory beyond the median calculation of the K epochs is provided by λ. Optimal results [20] were obtained using a buffer size of *K* = 236 and λ = 0.99 corresponding to a memory of several minutes (the effect of a single value decays to 1 % in about 15 min for *λ* = 0.99). In our study, features were calculated for 10-s epochs and baseline update was performed every minute. This approach differs from the approach used by Logesparan et al. [20] who used an epoch duration and update interval of both 1 s. To save computational time, a 1 min update interval was used since feature normalization has to be computed online for each of the 103 features and for each of the 19 channels. Because of the aforementioned difference in update interval and epoch duration, different values for *λ* and *K* had to be used. To match the same original decaying rate, a value of *λ* = 0.72 had to be used instead of 0.99. Similarly for a buffer size within the optimal range (236 s and above), a buffer size of *K* = 50 epochs (with 50 % overlap) was used, corresponding to the value 250 used by Logesparan.

### Novelty detection using principal component analysis

In contrast to MDM, where *F*_bsl_ is calculated using the 50 most recent epochs (Eq. 1), Novelty-MDM applies a procedure to select epochs for calculating *F*_bsl_. Instead of the most recent 50 epochs, the 50 most recently selected epochs were used to calculate *F*_bsl_. Epoch selection was performed by a novelty detector based on principal component analysis (PCA) [16]. This PCA model was trained on the epochs in the actual baseline buffer to detect non-similar epochs.

### Principal component analysis

PCA is a statistical procedure that uses an orthogonal transformation $T = XW$ to map a set of feature vectors **X** with correlated variables (EEG features) into a set of new uncorrelated variables called principal components [17]. These principal components are then sorted so that the first component accounts for the greatest amount of variance, the second component for the second amount of variance, and so on. Dimensionality reduction is performed by retaining only the first L components: $T_{\text{L}} = XW_{\text{L}}$. The original observations can now be reconstructed using only the first L components: $X_{\text{L}} = T_{\text{L}} W_{\text{L}}^{T}$. The total squared reconstruction error $E_{\text{rec}} = X - \left. {T_{\text{L}} W_{\text{L}}^{T} } \right\|_{2}^{2}$ can be used as a novelty measure. The idea behind this novelty detector is that the reconstruction error will be small for observations that originate from the same distribution as the observations *X* used to identify the principal components W. Larger reconstruction errors are expected for observations from another distribution.

### Novelty-MDM implementation

Novelty-MDM is performed per EEG channel separately, starting with the manually selected initial buffer of non-seizure EEG epochs. This buffer is represented by a matrix where each row represents an epoch and each column one of the 103 calculated EEG features. Due to differences in magnitude between different features, each feature is first normalized by subtracting its mean and subsequently dividing it by its standard deviation. Subsequently, the normalized matrix is used to calculate the PCA model. The ‘expected reconstruction error’ is calculated using a leave-one-out (LOO) procedure. Excluding one epoch at a time, a PCA model is trained on the remaining feature vectors, after which the reconstruction error is calculated for the left-out epoch. This is then repeated for each epoch, and the average reconstruction error of this LOO procedure is used to determine a threshold to classify new epochs as similar or novel. If the reconstruction error of a new epoch exceeds Tr times the expected reconstruction error, the epoch is classified as novel. Tr = 2 and L = 5 values were chosen heuristically. In case of Tr, the reconstruction error usually ranged between 1 and 2 times the expected reconstruction error. For artifactual epochs, the reconstruction was much larger (in the range of 20–200 or even above). Regarding the number of principal components, inclusion of more than 5 did not relevantly change the calculated reconstruction errors. This is to be expected because the amount of variance each component accounts for decays exponentially.

### Feature baseline correction

Feature baseline correction (FBC) is an EEG feature normalization method introduced by our group [3] which is performed by subtracting a normalization factor *N* from a raw feature value *F*:

2 $$F_{N} = F - N$$

with

3 $$N = A \times F_{\text{bsl}} + B$$

where *A* and *B* are parameters estimated from the training data set. A more detailed description of FBC can be found in our recent paper [3].

### Seizure detection framework

**Fig. 1:**
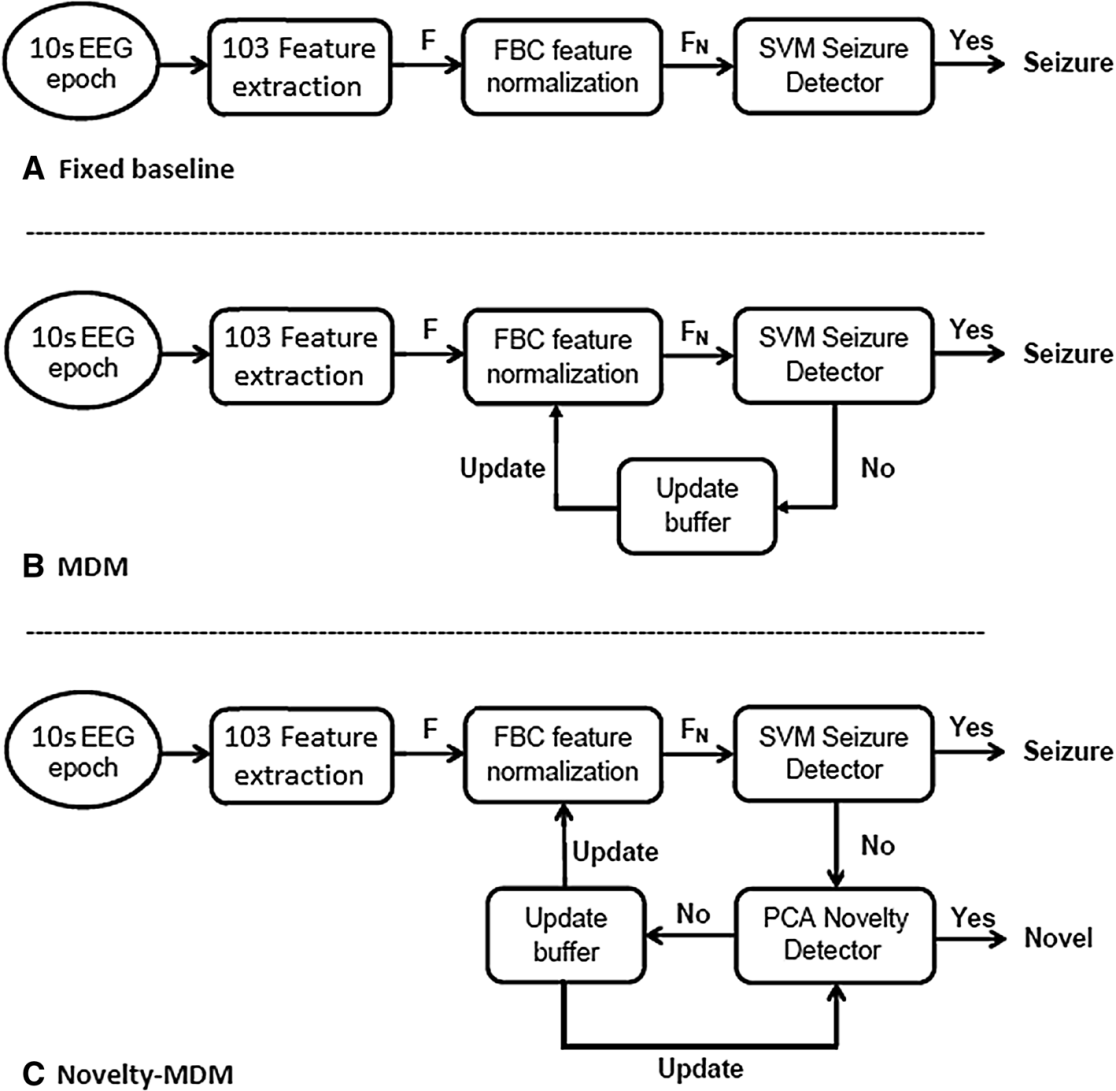
Schematic overview of SVM-based seizure detection framework with three different normalization methods. **a** Normalization constants derived from a fixed and manually selected EEG baseline segment. **b** Dynamic feature normalization using MDM. **c** Dynamic feature normalization using Novelty-MDM

The different seizure detection frameworks evaluated in this study are illustrated in Fig. 1. For each single EEG channel epoch, a set of 103 features is computed. These features are then normalized according to Eq. 2, after which the normalized feature vector is classified by an SVM classifier as either seizure or non-seizure.

Figure 1a illustrates the use of a fixed baseline segment to calculate *F*_bsl_. Figure 1b illustrates MDM, with the trivial exception that feature vectors successfully classified as seizure are excluded from the baseline. The Novelty-MDM approach is illustrated in Fig. 1c. With Novelty-MDM, feature vectors classified as non-seizure are subsequently classified by the novelty detector as either ‘Novel’ or ‘Non-Novel’. Only feature vectors classified as ‘Non-Novel’ are incorporated into the baseline buffer. Using the median decaying memory approach, a new set of normalization factors is calculated based on the updated baseline buffer. Based on this updated baseline buffer, a new novelty detector is calculated. The updated normalization factors and novelty detector are then applied to subsequent epochs.

### SVM seizure detection performance evaluation

The SVM seizure detector was trained on a randomly chosen subset of epochs from a set of routine EEG registrations from 39 neonatal patients. This classifier was readily at our disposal and, although it was trained on neonatal EEG, can also successfully be used for seizure detection in adults [10]. Because the random selection of the training data set may introduce variance in performance metrics, Monte-Carlo simulations were performed by training 25 classifiers trained on 25 random training subsets. Seizure detection performance was subsequently evaluated for these 25 classifiers using the test EEG dataset of 17 ICU patients. The average and standard deviation of the performance per patient allow for statistical testing, to determine differences between the three seizure detection frameworks. From our ICU dataset, the 17 patients with seizures were used to evaluate seizure detection performance using the 3 different feature normalization methods: (Fixed baseline (FB), MDM, and Novelty-MDM.

The seizure detection procedure was applied to each channel separately. If a seizure was detected in at least one channel, the complete epoch was classified as seizure.

To evaluate classifier performance, an ROC curve was obtained per EEG registration by plotting sensitivity versus specificity for all possible detection thresholds [11]. The area under this curve (AUC) was then used as a measure of the performance. AUC range is between 0.5 for random and 1 for perfect classification. AUC values were calculated per patient, and final performance measures were obtained by taking the average of the 25 AUC values. Group differences between the 3 seizure detection frameworks were tested for statistical significance using the nonparametric Wilcoxon signed rank test. Differences in performance per patient were tested for statistical significance using a paired *t* test. *p* values below 0.05 were considered statistically significant.

## Results

**Fig. 2:**
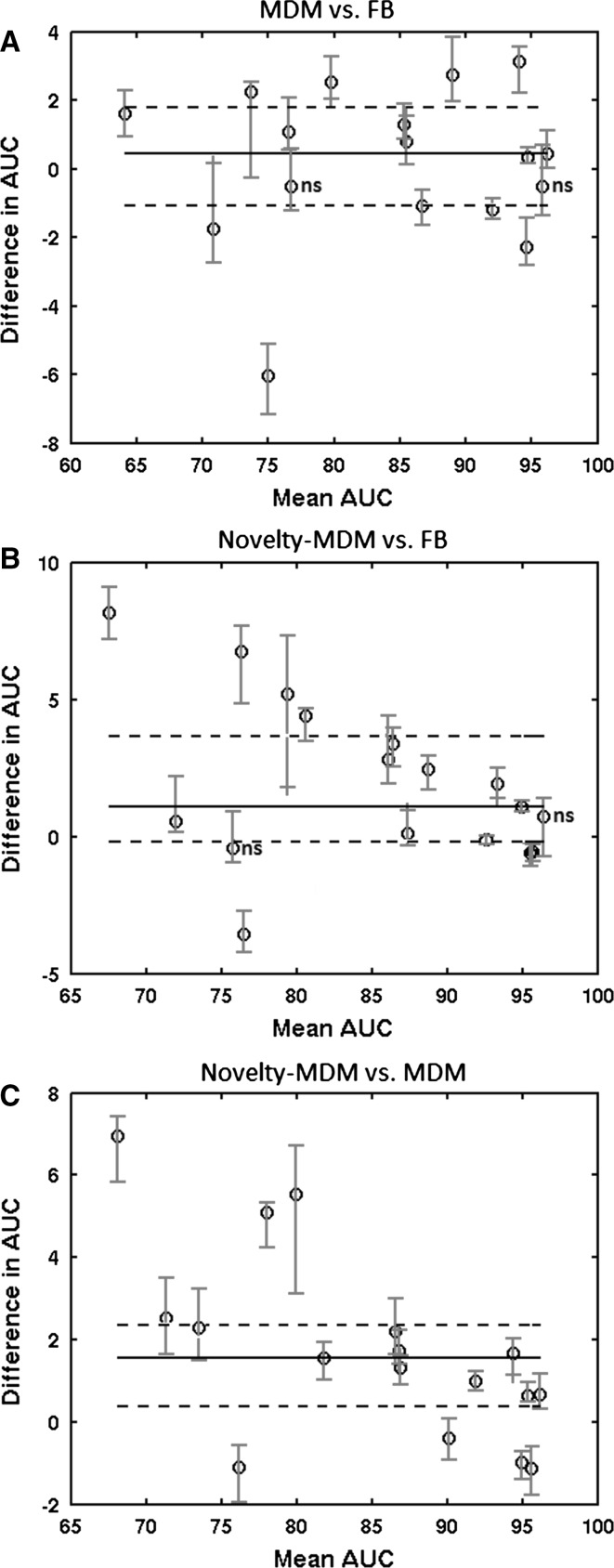
Mean difference plots of the AUC values per patient. Mean AUC values of two methods are plotted against their difference. Each *circle* represents the median value of the 25 Monte–Carlo simulations, and each *red bars* indicate the corresponding 25 and 75 percentile values.* Horizontal lines* indicate the group median (*broken line*), 25 and 75 percentile values (*solid line*). **a** Group difference MDM versus FB (*p* = 0.27). **b** Group difference Novelty-MDM versus FB (*p* = 0.015). **c** Group difference Novelty-MDM versus MDM (*p* = 0.0065). Non-statistically differences are indicated with* ns* (colour figure online)

**Fig. 3:**
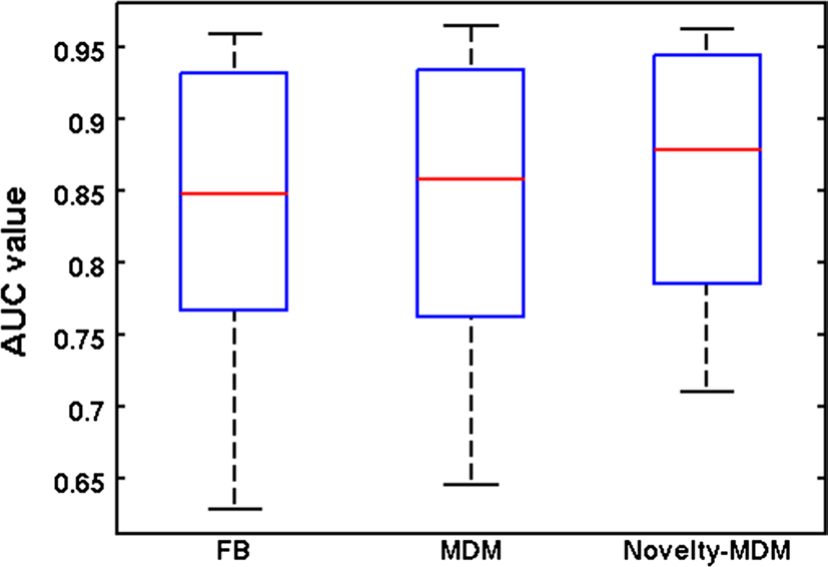
*Boxplots* describing the AUC value distributions for each normalization method

Looking at each individual patient, seizure detection performance was highest for Novelty-MDM in 8/17 patients, highest for MDM in 4/17 and highest for FB in 3/17 patients. For a single patient, all three methods resulted in the same performance and for another single patient either FB or Novelty-MDM performed best. The Bland–Altman plots in Fig. 2 show performance differences between all three methods by plotting method to method performance difference versus their average performance. Specific AUC value distributions for each of the 3 normalization procedures are shown in Fig. 3. Average AUC values per patient and per normalization method can be found in the appendix.

### MDM versus FB

To compare MDM versus FB feature normalization, Fig. 2a shows MDM minus FB AUC values versus their mean ((MDM + FB)/2) per patient. Ten patients showed improved performance, 5 patients decreased performance and for the remaining 2 patients, no statistically significant difference was found. In the complete group, MDM performance was not significantly different from FB (*p* = 0.27, Wilcoxon signed rank test).

### Novelty-MDM versus FB

Overall, Novelty-MDM was significantly better compared to FB (*p* = 0.015). Figure 2b shows that Novelty-MDM achieved higher performance for 11 patients, lower performance for 4 and equal performance for the remaining two patients.

### Novelty-MDM versus MDM

**Fig. 4:**
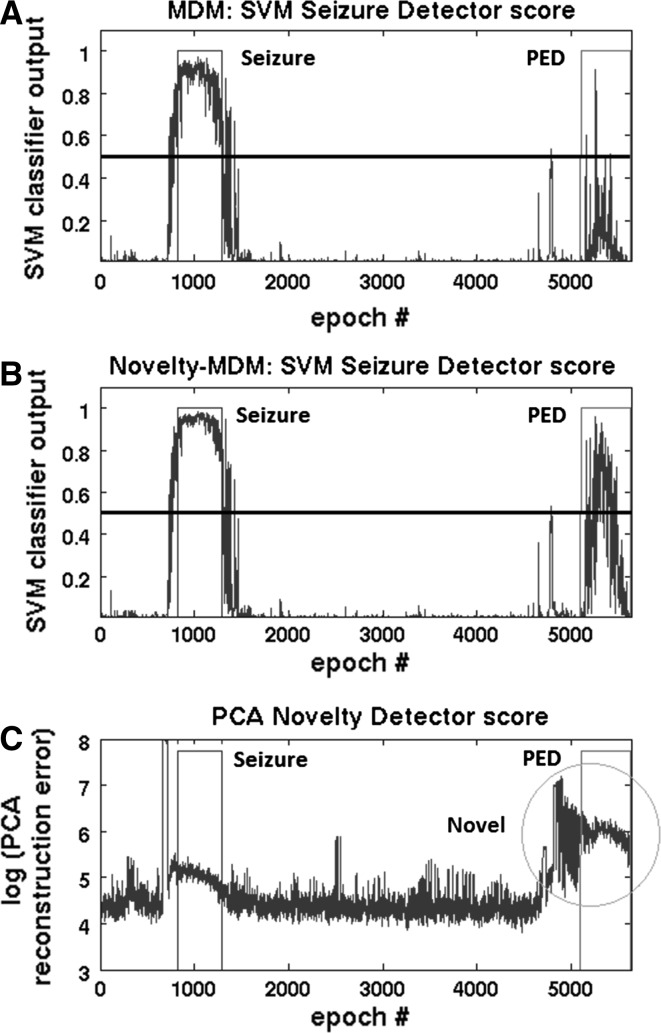
SVM classifier output for MDM (**a**) and Novelty-MDM (**b**), a detection threshold of 0.5 was used as indicated by the* horizontal lines*. The novelty detection score (reconstruction error) is shown in subplot (**c**). The *green rectangle* indicates an episode with seizures; the *red rectangle* indicates an episode with PED. The *yellow circle* indicates an episode of epochs that are classified as novel. The first half of this episode contains numerous electrode artefacts and the second half contains PED. Consequently, Novelty-MDM does not update the baseline during this episode because the artifactual and PED epochs were classified as Novel (colour figure online)

The numbers presented so far showed that both MDM and Novelty-MDM outperform FB. Patient specific Novelty-MDM versus MDM differences in classification performance are shown in Fig. 2c. Overall, Novelty-MDM performed better than MDM (*p* = 0.0065). Higher performance was found in 13 patients and lower for the remaining 4 patients. Detailed evaluation of the latter four cases revealed that this lowered performance was due to the presence of periodic epileptiform discharges (PED) and can be explained by the illustration in Fig. 4. It shows that a correctly detected seizure was followed by an episode with PED. Novelty-MDM classified more epochs as seizure compared to MDM. This is because at the start of the PED episode, MDM included these epochs into the baseline buffer, whereas Novelty-MDM did not. As a result, in case of MDM, the subsequent epochs containing PED were more similar to the baseline, and consequently, more likely to be classified as non-seizure. Novelty-MDM, on the other hand, excluded these epochs with PED because they were marked as ‘novel’ as can be seen in the bottom plot. As a result, the subsequent PED epochs were less similar to the baseline and consequently became more likely to be classified as seizure.

## Discussion

### Dynamic feature normalization

To correct for changing EEG characteristics and differences between patients, various feature normalization methods have been evaluated by Logesparan et al. [20]. Best performance was achieved by feature normalization using the median decaying memory (MDM) method. MDM uses the median feature value of a sliding baseline buffer for normalization. However, during long-term EEG monitoring, especially in an intensive care unit, seizures, artefacts, epileptiform activity, and other abnormal EEG patterns can be numerous. These patterns might contaminate the baseline buffer and by that, reduce seizure detection performance. Our study provides evidence that MDM is not robust enough for reliable dynamic feature normalization during long-term ICU EEG monitoring. We conclude this from the observation that in our 17 patients, MDM did not result in overall better performance compared to feature normalization with a fixed baseline. However, application of our newly introduced Novelty-MDM approach did result in an overall increase in performance both with regard to FB and MDM normalization. Novelty-MDM is based on a selection procedure to prevent erroneous epochs being included into the baseline buffer thereby improving feature normalization and subsequent seizure detection.

Although Novelty-MDM resulted in better performance for the majority (11/17) of patients, in four patients performance was lower compared to MDM. In three of these four patients, the decrease was due to false detection of epochs containing periodic epileptiform discharges (PED). This finding can be explained by the different behaviour of MDM versus Novelty-MDM. In the presence of PED, whose feature values are between background EEG and seizure EEG activity, MDM includes the PED epochs into the baseline buffer making it more ‘seizure like’ and thereby hampering seizure detection. Novelty-MDM, on the other hand, rejects these PED epochs and in this way prevents contamination of the ‘background EEG’ baseline buffer. As a result, MDM becomes less sensitive but more specific in the presence of PED whereas Novelty-MDM becomes more sensitive but less specific. However, the presence of PED does not necessarily result in lower performance for Novelty-MDM. Actually, better performance was found in four other patients (patient 6, 7, 10 and 17). Whether the presence of PED results in higher or lower AUC values might depend on the relative number of seizure/PED epochs, their occurrence in time relative to each other and the severity of the epileptiform activity, i.e. how closely they resemble true seizure activity. This illustrates that our current seizure detection algorithm is not always accurate enough to distinguish between epileptiform and seizure activity. To improve on this, more sophisticated algorithms are needed [28]. However, the underlying problem is the fact that seizure detection is approached as a two-class problem instead of a multi-class problem (non-seizure, seizure, epileptiform activity, and possibly other EEG phenomena). A restriction with regard to this will be that even an EEG expert cannot always make a clear distinction between epileptiform and epileptic activity [25]. This fundamental issue will always remain and by that limit automated reliable seizure detection.

### Implications, limitations and future research

This paper focused on challenges met in robust dynamic feature normalization applied in automated seizure detection algorithms. So far, MDM feature normalization using a sliding baseline did not take into account the presence of EEG patterns that might corrupt feature normalization. Our results have shown that a non-selective way of baseline update can have a negative effect on classification performance. Baseline epoch selection using a PCA-based novelty detector is a candidate solution to this problem. In cases where Novelty-MDM resulted in lower performance, it became clear that this was due to epochs containing epileptiform activity. Future research should focus on distinguishing epileptiform EEG from epileptic EEG. In the setting of seizure versus non-seizure classification, detection of epileptiform activity, in particular PED, is considered false detections. However, PED detection could also be considered useful because it is associated with a higher risk of seizures [4, 19]. Other sources that may cause lower detection performance, apart from electrode failure, muscle and movement artefacts, are patterns that are rhythmic in nature but do not represent seizure activity. Two of our patients had long-lasting periods of frontal intermittent rhythmic delta activity (FIRDA) which were falsely detected as seizure. If such EEG patterns persistently trigger false detections, future improvements incorporating these patterns into the SVM seizure detection algorithm as non-seizure would be of great value. Moreover, incorporating patient specific seizure information during online monitoring has proven to improve seizure detection performance [14, 27, 31]. Further research could be of great value when focussed on both incorporating various types of patient specific information such as seizure and background EEG data, as well as EEG patterns that caused false detections.

## Electronic supplementary material

Below is the link to the electronic supplementary material.

Supplementary material 1 (DOCX 15 kb)
