# Supplementary material for: Improved epileptic seizure detection combining dynamic feature normalization with EEG novelty detection
Source: Med Biol Eng Comput. 2016 Apr 6;54(12):1883–92. doi: 10.1007/s11517-016-1479-8 (PMC5104774; doi:10.1007/s11517-016-1479-8)
Supplement: Supplementary file 1 — Supplementary material 1 (DOCX 15 kb) [file 11517_2016_1479_MOESM1_ESM.docx]

| **FB** | **MDM** | **Novelty-MDM** |
| --- | --- | --- |
| 0.848 (0.023) | 0.863 (0.017) | 0.881 (0.016) |
| 0.958 (0.011) | 0.964 (0.008) | 0.952 (0.011) |
| 0.927 (0.005) | 0.914 (0.008) | 0.925 (0.006) |
| 0.950 (0.023) | 0.928 (0.026) | 0.944 (0.026) |
| 0.873 (0.027) | 0.902 (0.018) | 0.897 (0.021) |
| 0.945 (0.009) | 0.949 (0.009) | 0.956 (0.012) |
| 0.845 (0.035) | 0.855 (0.028) | 0.878 (0.022) |
| 0.870 (0.021) | 0.858 (0.022) | 0.871 (0.022) |
| 0.755 (0.036) | 0.769 (0.034) | 0.755 (0.037) |
| 0.733 (0.031) | 0.747 (0.028) | 0.795 (0.030) |
| 0.782 (0.024) | 0.809 (0.022) | 0.823 (0.020) |
| 0.771 (0.036) | 0.768 (0.035) | 0.822 (0.046) |
| 0.770 (0.044) | 0.711 (0.050) | 0.736 (0.041) |
| 0.629 (0.036) | 0.646 (0.029) | 0.711 (0.029) |
| 0.953 (0.029) | 0.955 (0.017) | 0.962 (0.020) |
| 0.924 (0.016) | 0.954 (0.008) | 0.944 (0.011) |
| 0.720 (0.057) | 0.707 (0.045) | 0.732 (0.051) |

**Table S 1.** Average (standard deviation) AUC values of the SVM classifier for each of the 3 normalization methods: Fixed Baseline (FB), Median Decaying Memory (MDM), Novelty Median Decaying Memory (Novelty-MDM). Statistically significant optimal values are highlighted in green.
